# Custom-Made 3D-Printed Implants as Novel Approach to Reconstructive Surgery after Oncologic Resection in Pediatric Patients

**DOI:** 10.3390/jcm10051056

**Published:** 2021-03-04

**Authors:** Giovanni Beltrami, Gabriele Ristori, Anna Maria Nucci, Alberto Galeotti, Angela Tamburini, Guido Scoccianti, Domenico Campanacci, Marco Innocenti, Rodolfo Capanna

**Affiliations:** 1 Department of Pediatric Orthopedic and Pediatric Orthopedic Oncology, Meyer Children Hospital, Viale Pieraccini 24, 50139 Florence, Italy; gabrieleristori@gmail.com (G.R.); annamarianucci90@gmail.com (A.M.N.); albertogaleotti91@gmail.com (A.G.); 2 Department of Pediatric Oncology, Meyer Children Hospital, Viale Pieraccini 24, 50139 Florence, Italy; angela.tamburini@meyer.it; 3 Department of Orthopedic Oncology, Careggi Hospital, Largo Piero Palagi 1, 50139 Florence, Italy; scocciantig@aou-careggi.toscana.it (G.S.); campanaccid@gmail.com (D.C.); marcoinnocenti1212@gmail.com (M.I.); 4 Orthopedic Department, University of Pisa, Via Paradisa 2, 56124 Pisa, Italy; rodolfo.capanna@unipi.it

**Keywords:** custom-made prosthesis, pediatric limb salvage, bone tumor, vascularized flap

## Abstract

Recently, custom-made 3D-printed prostheses have been introduced for limb salvage surgery in adult patients, but their use has not been described in pediatric patients. A series of 11 pediatric patients (mean age 10.8 years; range 2–13) with skeletal tumors treated with custom-made implants for the reconstruction of bony defects is described. Patients were followed up every 3 months. Functional results were evaluated by the Musculoskeletal Tumor Society Score (MSTS) for upper and lower limbs. The mean follow-up was 25.7 months (range 14–44). Three patients died after a mean of 19.3 months postoperatively—two because of disease progression and the other from a previous malignancy. Three patients experienced complications related to soft tissues. One patient required device removal, debridement, and antibiotic pearls for postoperative infection. Partial osseointegration between grafts and host bone was observed within a mean of 4 months. At the final follow-up, mean MSTS score was 75%. 3D prostheses may yield biological advantages due to possible integration with the host bone and also through the use of vascularized flaps. Further research is warranted.

## 1. Introduction 

In pediatric patients who are affected by malignant bone tumors, recent progress of oncologic medical therapies has made primary amputation increasingly rare [1]. Massive resections, which are often performed, present manifold challenges in limb reconstruction. Reconstruction systems are often designed for adults and have to be readapted to children, who are smaller. Additionally, skeletally immature patients have high functional–biomechanical demands. Furthermore, failure to heal in an anatomically correct position or resection of the growth plate may lead to joint incongruence and growth disturbances [2]. 

Over the past several decades, various reconstructions have been extensively used for limb salvages in the pediatric population [3,4]. Conventional prostheses can provide immediate support, a rapid return to weight-bearing, and a valid articular surface replacement, but they are associated with frequent complications (aseptic loosening, infection, and periprosthetic fracture). Despite the use of modular systems, they may not fit perfectly in children. Moreover, they are unable to accommodate the patient’s growth, which is the reason why expandable prostheses have been introduced with varying degrees of success [5,6]. Bone allograft can supply immediate structural support as well as an anchor for reattachment of ligaments and muscles. Allografts can also be used in articular reconstruction, but their survival is put to the test in patients with high functional demands [7]. Moreover, they have to be manually carved to precisely fit the defects, and this process is usually time-consuming and laborious. Bone autograft has superior healing capability through osteogenesis, osteoconduction, and osteoinduction, alone or in combination [8]. However, it requires invasive collection, and it can hardly be used in articular reconstructions [9]. Treated autograft has been proposed as another solution. It consists of a replantation after extracorporeal irradiation, heat pasteurization, or freezing with liquid nitrogen of the resected specimen [10]. More studies and longer follow-up are required to confirm the method’s effectiveness. Allograft prosthesis composite is a bone massive allograft resurfaced by a conventional prosthesis, which overcomes the articular collapse of conventional bone massive allograft and maintains the enthesis and mechanical properties of homologous bone [11]. However, this solution is challenging with the ongoing growth of the patient. Furthermore, the use of allograft prosthesis composite is associated with a high rate of long-term complications. Each solution has its own advantages and limitations, and the choice must be carefully weighed on an individual basis.

Recently, custom-made 3D-printed prostheses have been introduced for limb salvage surgery in adult patients. They are based on individual digital planning procedures, rapid prototyping with 3D-printing technology, and titanium alloy implants that are proven to be effective [12].

We believe this technology might be well adapted to pediatric patients, allowing complex reconstructions that may not have been possible until today. However, no study in the literature focuses specifically on the application of customized implants in this population. The objective of this study was to describe a series of patients younger than 14 years who received a 3D-printed, custom-made prosthesis and to report the follow-up results.

## 2. Experimental Section

From December 2016 to June 2019, 11 pediatric patients (6 males and 5 females) with primary malignant bone tumors received resection surgery and reconstruction with a custom-made 3D-printed implant in our hospital. Inclusion criteria for the study were the presence of a primary malignant bone tumor, absence of secondary metastases, and age less than 14 years. Furthermore, the tumor had to be removed close to the physis, and there had to be a need for either anatomic reconstruction or an osteoarticular reconstruction without viable surgical alternatives. The mean age at surgery was 10.8 years (range, 2–13). Histology included Ewing’s sarcoma in 6 patients, osteosarcoma in 4 patients, and rhabdomyosarcoma in 1 patient. The anatomic site involved was the humerus in 2 patients, scapula in 1 patient, hemipelvis in 2 patients, femur in 2 patients, tibia in 2 patients, calcaneus in 1 patient, and radius in 1 patient. For each patient, oncological staging was detected, and all tumors were located at the primary site without distant metastases. In this study, we performed a retrospective review of this series of patients. 

Before undergoing operative treatment, all patients completed the diagnostic path with radiography, computed tomography (CT), and contrast-enhanced magnetic resonance imaging (MRI). Each patient underwent CT-guided needle biopsy to obtain a pathological diagnosis. Moreover, an oncological evaluation was performed to plan the appropriate medical treatment and radiotherapy to follow surgery. The resection area was then clearly defined.

Once the patients’ parents provided their permission, surgery was planned, and the manufacturing process of a custom-made implant was initiated. Firstly, a computer-aided design model of the affected bone segment was designed using the patient’s CT-scan data. The level of osteotomies was decided by the surgeon, and then the custom implant was designed to match the segmental gap after removal of the diseased tissue. The implant design incorporated the specifications decided on by the surgeon in order to secure the device to the host bone (i.e., screws, stems, and plates). If necessary, one or more grooves were placed to accommodate a bone graft to facilitate the integration between the metal device and the residual host bone. Custom implants were manufactured employing a modern 3D-printing technique [13]. Manufacturing time was approximately 4 weeks in all cases. All implants were made of cobalt-chromium-molybdenum alloy or porous titanium alloy with titanium niobium nitride coating. Customized instrumentation was provided in more complex procedures. The quality of the surgical margin was indicated in accordance with the Residual Tumor (R) Classification, with R0 defined as no residual tumor, R1 as microscopic residual tumor, and R2 as macroscopic residual tumor [14]. 

Postoperatively, all patients were treated with physiotherapy for functional recovery. Orthotics were provided, if necessary. All patients were entrusted to the oncologic department for the continuation of medical adjuvant treatment. Patients underwent follow up every 3 months with an outpatient control in order to recognize the oncological outcome in terms of disease progression (local recurrence or distant metastasis). Plain radiographs, local CT scan, local MRI, chest CT scan, and abdominal and lymph-node ultrasound were used for follow-up purposes. Complications that were defined on the basis of Henderson classification [15] included the following: soft tissue failure (musculo-ligamentous deficiency or wound dehiscence), aseptic loosening, structural failure (implant breakage, graft fractures, or peri-prosthetic fractures), infection, and tumor progression. Another possible complication included graft–host nonunion, discerning delayed union (less than 1 year) from nonunion (more than 1 year) [16]. Pediatric-specific failures, such as physeal arrest or dysplastic joint, resulting from articulation with implant or graft, were another complication for consideration. The re-operation rate due to any of the listed causes has been further defined.

The functional results were evaluated by the Musculoskeletal Tumor Society Score (MSTS) for upper and lower limbs [17]—in particular, pain, articulation recovery, and residual etherometry. Based on the tumors’ features and staging, we treated patients with a multidisciplinary approach following the conventional protocols. In addition to surgery, all patients received neoadjuvant and adjuvant chemotherapy, except for 1 patient with a low-grade periosteal osteosarcoma. One patient received adjuvant radiotherapy. Patient characteristics are summarized in Table 1.

In accordance with the legislation of the country where the study was performed, ethics committee approval was not obtained, as the study was purely observational. 

## 3. Results

**Figure 1:**
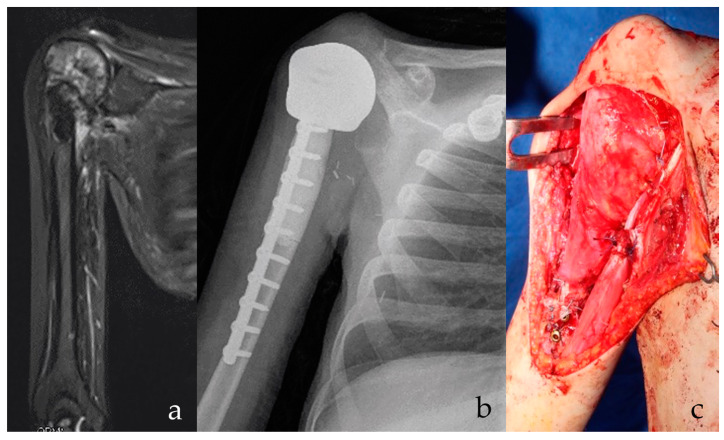
(**a**) Osteosarcoma of the proximal humerus in Li Fraumeni Syndrome is shown on radiography. Possible infected biopsy tract. Patient had prior brain medulloepithelioma with residual parethic lower limb. Stable and effective shoulder function are essential for everyday activities. (**b**) After intraarticular proximal humerus and deltoid resection, a custom-made, 3D-printed anatomical proximal humerus reconstruction with diaphyseal antibiotic cement is implanted. At 15 months follow-up, the implant is stable. (**c**) A latissimus dorsi rotational flap provides for functional recovery of the deltoid and offers a biological barrier to infection. A stable and effective implant is shown, with low risks of secondary infection.

**Figure 2:**
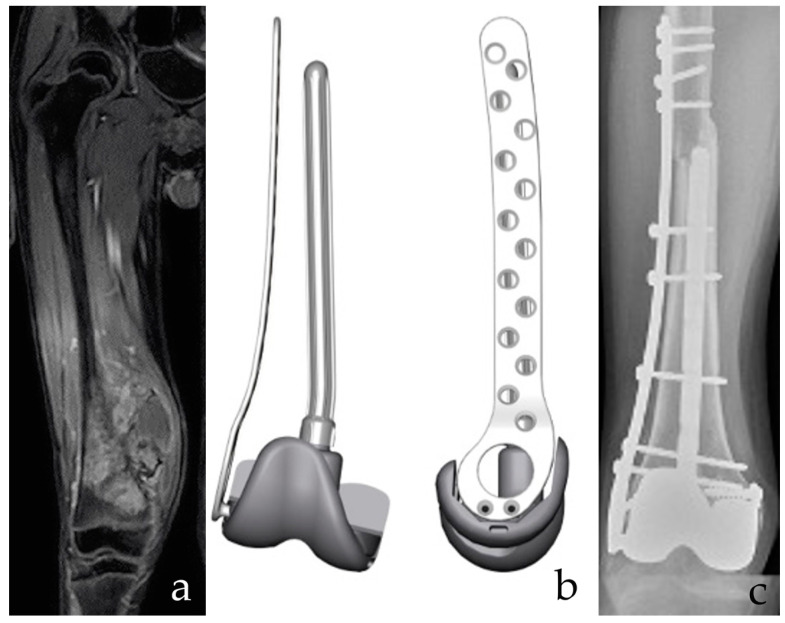
(**a**) A 12-year-old male patient with osteosarcoma of the distal femur after neoadjuvant chemotherapy. Intrarticular resection. (**b**) The planning of a custom-made, 3D-printed plate combined with a resurfacing prosthetic implant and intramedullary stem. Both the prosthetic surface and the intramedullary stem are removable in case of failure of the allograft, with bone stock retaining for conventional Allograft Prosthesis Composite (APC). (**c**) APC with bone massive allograft of the distal femur. Bone stock reconstruction and sparing of the proximal tibia growth plate are provided. The collateral and cruciate ligaments are functional. Postoperative imaging confirms fusion of the osteotomy line and stable implant positioning.

The mean follow-up time was 25.7 months (range, 14–44). All but one patient underwent an initial surgery based on tumor resection with wide margins reported to the histological intraoperative analysis; in a single patient, an R1 microscopic residual tumor was reported. In nine patients with a difficult anatomical location, the 3D-printing technology described above was used to create a custom-made cutting guide to improve the osteotomies. Reconstruction with a custom-made implant was then conducted, which included a prosthesis in seven patients, an anatomical plate in three patients, and a prosthetic element linked to an anatomical plate in one patient (Table 1, Figure 1 and Figure 2). 

Great importance was given to bone graft augmentation. Six patients received an allograft (strut or corticocancellous bone graft), and three patients received a vascularized fibular flap performed by a microsurgical team. Additionally, the soft tissue coverage required a primary muscular or a myofascial vascularized flap in five cases. Three cases were reconstructed with a titanium custom-made prostheses and structural antibiotic cement (one case) or soluble antibiotic pearls (two cases). This approach was utilized in order to minimize the infection rate in patients who were at high risk of infection.

One complication was related to the surgery—a venous thrombosis of a musculocutaneous free flap in the calcaneal region recorded in the first 12 h after surgery, which was successfully revascularized by the microsurgeon. However, the patient developed a deep infection and was subsequently treated with implant removal and antibiotic pearls. The patient is waiting for a new biological reconstructive option with a persistent limb salvage option.

Two patients died 15 and 25 months postoperatively because of disease progression (local recurrence and pulmonary metastases). Another patient died 18 months after surgery due to an unrelated cause: a previous malignant medulloepithelioma in Li Fraumeni syndrome. Two patients experienced disease progression with pulmonary metastases after 6 and 20 months, and they are currently continuing chemotherapy. The remaining six patients (55%) are alive and continuously free from disease. At follow-up, three patients (25%) experienced local complications of the soft tissue—in particular, wound dehiscence. Two patients underwent a subsequent musculocutaneous flap. There was one case of limb length discrepancy (treated by contralateral transient epiphisiodesis), with no dysplastic joint or physeal arrest, even though the growing phase has not concluded and follow-up must be continued. Partial osseointegration between various grafts and host bone has been radiographically observed within a mean of 4 months, while complete osseointegration occurred in an average of 6 months. 

Functional outcomes were generally satisfactory (Table 2), with a mean score according to the MSTS 93 system of 75%. At the last follow up, only two patients reported a fair MSTS score (32%), one due to a restricted range of motion (even though the implants were considered stable and functionally effective), and the other had a fair score after device removal (with another limb salvage procedure scheduled for this patient). The remaining patients showed a good MSTS score (ranging from 56% to 75%), except for two cases reporting excellent outcomes (93%).

## 4. Discussion

The development of custom-made implants represents the latest innovation in the field of limb salvage surgery after malignant bone tumors [18,19]. However, the application of this technology in the pediatric population has not been described until now. Specifically, the sizes of the implants can be exactly proportioned to the sizes of resected bone. This represents a significant advantage as compared with conventional prostheses and even modular ones. Since those implants are designed for the bone of adult patients, they have inherent problems of mismatching or oversizing, and consequent stress shielding, periprosthetic fractures, and aseptic loosening are always possible. Customized implants also offer an advantage with regard to bone massive allografts. The bone usually comes from adult donors and has to be manually carved to precisely fit the size of a pediatric patient. In contrast, the accurate fitting of a 3D-printed implant to the host bone leads to high primary stability, which represents a biomechanical advantage.

Furthermore, not only the size but also the shape of the implant is fundamental. In these terms, 3D implants could provide an articular surface, customized plate, or integrated systems plate-prosthesis. This permits preservation of the physeal plates avoiding growth arrest that may result in etherometry, angular deformities, or joint incongruity. Moreover, with viable physis, expandable implants are not necessary, and complications associated with lengthening malfunction are avoided.

3D-printed custom-made implants allow for optional sites of insertion for tendons, ligaments, or capsules. 3D-printed implants also have the advantage of saving a portion of joint surfaces; they can therefore be used as uni-compartmental and hemi-arthroplasty procedures. Furthermore, 3D-printed implants are useful in the reconstruction of bone segments with complex anatomy, in which conventional prostheses are not available, such as in the pelvis, tarsal bone, clavicle, or scapula [12,20,21].

The possibility of growth accommodation of the implant is another important feature for pediatric patients. 3D-printed custom implants can also include removable devices (in particular anatomical plates) that would function to support or provide temporary mechanical reinforcement, which could later be removed when their function is deemed to be complete or in case of revision. These devices can also be placed, if necessary, over a physis, providing a temporary epiphysiodesis without residual discrepancy if removed at the correct time.

Moreover, accurate resection is ensured by planning and—if provided—by the cutting guide. They are also effective for residual bone stock. In pediatric patients, it is important to achieve the least extensive, yet most useful, resection because they are candidates for future revision surgeries.

We paid close attention to biological aspects, aiming always to join the prosthetic element with bone or soft tissue vascularized flap. As shown above, all of our patients except for two received a vascularized flap. Vascularized flaps provide a setting rich in substrates and reparative tissue around the titanium prostheses [18,22], which may favor osseointegration.

Our overall results are satisfactory, with functional outcomes reporting an MSTS score of good or excellent in 87.5% of patients. The oncological results confirm that the procedure is safe, allowing resection with wide margins. Nevertheless, two patients died as a result of progression of the primary disease. The complications reported are linked in particular to the soft tissue coverage. Resections are usually wide, including the removal of superficial myocutaneous region, so the implant could cause skin problems. In our series, three patients (27%) experienced dehiscence of the surgical wound and needed revision surgery, with implant removal in one case of deep infection.

Our study had several limitations. Firstly, the wide variability of anatomical locations involves a heterogeneous group of patients who are difficult to compare, and there was a lack of control for comparison purposes between different reconstructive solutions. Secondly, the small sample size due to the low incidence of sarcoma did not allow sufficient power to explore the advantages of the procedure. Thirdly, we are only able to provide preliminary results to introduce the technique into the pediatric population.

In this study, custom-made prostheses to reconstruct bone segment after oncological resection are described in a pediatric population for the first time. The preliminary follow-up results proved the safety and effectiveness of the procedure, although this single-center study was small in scale and the follow-up period was short. The wide versatility of the system has the ability to adapt to various sites and to respect the physis. We believe that this prosthesis offers biological advantages, as it may integrate with the host’s bone or, if used, with the soft tissue vascularized flap. Furthermore, the 3D-printing technology allows manufacturing of the prosthesis in a relatively short time. However, although our initial results are promising, further studies with more patients and longer follow-up time are needed to confirm our preliminary conclusions.

## Figures and Tables

**Table 1:** Study population.

Patient	Sex	Age	Anatomical Site	Histology	Concomitant Therapy	Custom Implant	Quality of the Surgical Margin	Bone/Spacer	Soft Tissue
1	M	13	Humerus	High grade Osteosarcoma	Neoadjuvant and adjuvant CHT	Proximal humerus prosthesis with integrated plate	R0	Structural antibiotic cement	Latissimus dorsi rotational flap for deltoid region, vascularized and innervated
2	F	13	Scapula	Ewing sarcoma	Neoadjuvant and adjuvant CHT	Scapular prosthesis	R0	None	Latissimus dorsi rotational flap vascularized and innervated for subscapularis recovery
3	M	13	Pelvis	Ewing sarcoma	Neoadjuvant and adjuvant CHT	Ileum prosthesis	R0	Bone chips	Fascia lata rotational flap
4	F	11	Tibia	Ewing sarcoma	Neoadjuvant and adjuvant CHT	Anatomical Plate	R0	Massive allograft + fibular vascularized flap	Medial gastrocnemius rotational flap after scar slough
5	M	8	Tibia	Low Grade Osteosarcoma	None	Anatomical Plate	R0	Massive allograft + fibular vascularized flap	Medial gastrocnemius rotational flap after scar slough
6	F	13	Humerus	High grade Osteosarcoma	Neoadjuvant and adjuvant CHT	Subtotal humerus prosthesis	R0	Bone chips + fibular vascularized flap	None
7	M	13	Femur	High grade Osteosarcoma	Neoadjuvant and adjuvant CHT	Distal femur prosthesis with integrated plate	R0	Massive bone allograft	None
8	M	9	Calcaneus	Ewing sarcoma	Neoadjuvant and adjuvant CHT with RHT	Calcaneus prosthesis	R1	Non vascularized fibular strut autograft	Antero-lateral fascio-cutaneous free flap
9	M	13	Hemipelvis	Ewing sarcoma	Neoadjuvant and adjuvant CHT	Ileum prosthesis	R0	Antibiotic soluble pearls	Fascia lata rotational flap
10	F	13	Subtotal radius	Rhabdomyo sarcoma	Neoadjuvant and adjuvant CHT	Osteoarticular radius	R0	Antibiotic soluble pearls	None
11	F	2	Subtotal femur	Ewing sarcoma	Neoadjuvant and adjuvant CHT	Anatomical Plate	R0	Structural bone massive allograft	None

Abbreviations; CHT, chemotherapy; RHT, regional hyperthermia.

**Table 2:** Postoperative outcome.

Patient	MSTS	Postoperative Complications	Timing of Physical Recovery after Surgery
1	32%	Restricted range of motion	3 months
2	93%	None	3 months
3	85%	None	5 months
4	75%	Wound dehiscence	6 months
5	75%	Wound dehiscence	6 months
6	93%	Proximal wound dehiscence	4 months
7	85%	Stiff knee	5 months
8	32%	Postoperative venous thrombosis of the musculocutaneous free flap	6 months
9	75%	None	5 months
10	80%	None	5 months
11	90%	Stiff knee	5 months

Abbreviation: MSTS, Musculoskeletal Tumor Society Score.
